# Surgical Approaches for Prevention of Neuroma at Time of Peripheral Nerve Injury

**DOI:** 10.3389/fsurg.2022.819608

**Published:** 2022-06-27

**Authors:** Benjamin B. Scott, Jonathan M. Winograd, Robert W. Redmond

**Affiliations:** ^1^ Division of Plastic and Reconstructive Surgery, Massachusetts General Hospital, Harvard Medical School, Boston, MA, United States; ^2^ Wellman Center for Photomedicine, Massachusetts General Hospital, Harvard Medical School, Boston, MA, United States

**Keywords:** neuroma, peripheral nerve, nerve reconstruction, microsurgery, nerve injury and regeneration

## Abstract

Painful neuroma is a frequent sequela of peripheral nerve injury which can result in pain and decreased quality of life for the patient, often necessitating surgical intervention. End neuromas are benign neural tumors that commonly form after nerve transection, when axons from the proximal nerve stump regenerate in a disorganized manner in an attempt to recreate nerve continuity. Inflammation and collagen remodeling leads to a bulbous end neuroma which can become symptomatic and result in decreased quality of life. This review covers surgical prophylaxis of end neuroma formation at time of injury, rather than treatment of existing neuroma and prevention of recurrence. The current accepted methods to prevent end neuroma formation at time of injury include different mechanisms to inhibit the regenerative response or provide a conduit for organized regrowth, with mixed results. Approaches include proximal nerve stump capping, nerve implantation into bone, muscle and vein, various pharmacologic methods to inhibit axonal growth, and mechanisms to guide axonal growth after injury. This article reviews historical treatments that aimed to prevent end neuroma formation as well as current and experimental treatments, and seeks to provide a concise, comprehensive resource for current and future therapies aimed at preventing neuroma formation.

## Introduction

Neuroma is a frequent sequela of peripheral nerve injury that can become painful and disabling for the patient. First described in 1811 by Odier (1), neuroma is classified as a benign proliferation of neural tissue, containing all elements of the nerve sheath and nerve fibers, that occurs as a result of disorganized axon regrowth following nerve injury (2). Neuromas can occur after laceration, crush, blunt trauma, or chronic irritation to the nerve (3). While blunt trauma and/or chronic irritation can result in endoneurial and/or perineurial disruption thus leading to a neuroma-in-continuity, this review will focus on end neuromas which most commonly result after nerve transection injuries (4). Wallerian degeneration is initiated after nerve transection with the degradation of the axonal axoplasm followed by axonal cytoskeleton (5, 6). Schwann cell proliferation begins soon after and plays a key role in axonal regeneration, responsible for secretion of neurotrophic and neurotropic factors for axon regeneration as well as structural re-organization to facilitate axonal outgrowth (7). This axonal regeneration occurs in a disorganized fashion if no viable distal target exists, resulting in a tangle of axons and connective tissue that forms a bulbous neuroma (3, 8). Following limb amputation, where nerve transection occurs as part of the procedure, symptomatic end neuroma formation is common and is reported to result in residual limb pain in as many as 61%–74% of cases (9–11). Residual limb pain due to symptomatic end neuroma is a devastating complication of limb amputation, resulting in localized pain typically felt in the sensory territory of the affected nerve when aggravated by mechanical or thermal stimuli (11). This complication may result in decreased quality of life for the patient due to pain, inability to wear a prosthesis and psychological distress, and often requires surgical revision (12, 13). Although traction neurectomy, a process in which the nerve is pulled distally and transected sharply then allowed to retract into the limb stump, is no longer the surgical standard of care at time of both upper and lower extremity amputation, the technique continues to be performed by many surgeons (14–17). Because traction neurectomy alone leads to a high rate of post-amputation neuroma (18, 19). current clinical focus is more towards management of neuroma (20–22) rather than prevention of neuroma formation.

Surgical approaches to prevent formation of neuroma at the time of initial peripheral nerve injury have varied widely and include reducing and controlling inflammation, ablating the regenerative response, and providing a conduit for organized regrowth. A wide variety of treatments, including pharmacologic, massage, local injection, and surgical resection with and without reconstruction have been well studied (20, 23), however a comprehensive review of mechanisms to prevent neuroma formation *at time of injury* is lacking. This review will describe historical treatments to prevent end neuroma formation as well as current pre-clinical and clinical methodologies aimed at preventing neuroma formation at time of nerve injury (Table 1).

**Table 1:** Comprehensive literature review table summarizing surgical technique, author, year of publication, model of study, result, and suitability for clinical use.

Surgical Technique	Study	Year	Model	Result	Suitability for clinical use
Nerve implantation into adjacent tissue	Dellon et al. (26)	1984	Baboon	Parallel nerve fibers without evidence of neuroma when the nerve remained in muscle	This classic, simple technique may result in symptomatic neuroma more frequently when compared to methods that provide a conduit for active regrowth
	Low et al. (32)	2000	Rat	Implantation into vein results in smaller neuroma	
	Koch et al. (31)	2003	Rat	Implantation into vein results in 19%–37% reduction in size of neuroma	
	Sinis et al. (30)	2007	Rat	Implantation into muscle results in smaller neuroma	
	Prasetyono et al. (29)	2014	Rat	Implantation into vein results in absence of neuroma. Implantation into muscle results in smaller neuroma compared to control	
Pharmacologic inhibition	Guttman et al. (33)	1942	Rabbit	Formaldehyde (20%) and gentian violet (1%) inhibit neuroma formation, alcohol ineffective	Success varies with pharmacologic agent but is generally not suitable for clinical use due to high toxicity and/or inffectiveness
	Petropoulos et al. (34)	1961	Canine	Formalin (5%), alcohol (80%), and phenol inhibit neuroma formation	
	Nennesmo et al. (37)	1986	Mouse	Ricin prevents neuroma formation, concentrations >1 mg/ml resulted in death due to toxicity	
	Cummings et al. (36)	1988	Equine	Ricin induces cell body death and prevents neuroma. Doxarubicin inhibits axonal growth	
	Brandner et al. (35)	1989	Rat	Ricin prevents neuroma formation regardless of concentration	
	Kryger et al. (38)	2001	Rat	trkA-IgG (nerve growth factor inhibitor) decreases incidence of neuroma (38%) compared to controls (80%)	
Synthetic capping	Edds et al. (40)	1945	Rat	Methyl methacrylate caps inhibit axonal regeneration and neuroma formation	Synthetic caps may prevent neuroma formation but have high risk of foreign body reaction
	Swanson et al. (41)	1977	Rabbit	Caps ≥10 mm in length and of similar diameter to the nerve inhibit neuroma formation	
Autologous capping	Muehleman et al. (53)	1990	Rat	Epineurial caps result in smaller neuromas confined to area of nerve transection	Autologous caps do not prevent neuroma formation
	Yuksel et al. (55)	1997	Human	Epineurial grafts are significantly more effective in preventing neuroma pain compared to epineurial ligatures and flaps	
	Galeano et al. (56)	2009	Rat	Vein caps result in more preserved nerve architecture and smaller neuroma	
	Siemionow et al. (54)	2017	Rat	Epineurial caps reduce neuroma formation and Tinel sign	
Biomaterial capping	Marcol et al. (57)	2011	Rat	Chitosan caps reduce incidence and size of neuroma, no decrease in autotomy	Success of biomaterial caps varies by material, but may decrease axonal regeneration
	Agenor et al. (58)	2017	Rat	Hyaluronic acid/carboxymethyl cellulose caps decrease, but do not inhibit, axonal regeneration	
	Tork et al. (59)	2020	Rat	Porcine small intestinal submucosa caps increase axon to collagen ratio, decrease axon swirling, and reduce pain sensitivity	
	Hong et al. (60)	2021	Rat	Acellular nerve allograft caps ≥2.5 cm in length result in axonal regeneration arrest within allograft	
	Pan et al. (61)	2021	Rat	5 cm acellular nerve allografts limit axonal regeneration and lead to down-regulation of pain-associated genes within dorsal root ganglia	
Epineurial closure and fascicle cauterization	Battista et al. (43)	1981	Rat	Fascicle ligation decreases neuroma formation	Epineurial closure may reduce neuroma formation
	Martini et al. (44)	1989	Rat	Epineurial sleeves sealed with histoacryl glue decrease incidence of neuroma formation	
	Rahimi et al. (45)	1992	Rat	Epineurial sealing with radiofrequency coagulation only partially successful in preventing neuroma (44% vs. 77% of controls)	
Laser photocoagulation	Fischer et al. (47)	1983	Rat	No difference in incidence of neuroma after sciatic nerve transection with scalpel or CO_2_ laser	CO_2_ laser is ineffective at preventing axonal regeneration or neuroma. Nd:YAG laser transection reduces neuroma size and incidence.
	Hurst et al. (48)	1984	Rat	Conventional scissor transection of nerve is superior to CO_2_ laser transection	
	Montgomery et al. (50)	1985	Equine	Nerve transection with the CO_2_ laser at a power density of 3000 W/cm^2^ prevents neuroma formation	
	Haugland et al. (49)	1992	Equine	CO_2_ laser transection does not prevent axonal regeneration or neuroma formation	
	Menovsky et al. (51)	1999	Rats	Nd:YAG laser transection decreases neuroma size and adhesions when compared to sharp transection	
	Elwakil et al. (52)	2008	Rabbit	Nd:YAG laser transection reduced neuroma incidence (33%) compared to sharp transection (83%)	
Nerve conduit	Yan et al. (68)	2015	Rat	Nanofiber conduit decreases the weight ratio of neuromas after sharp transection	Nerve conduits result in more normal nerve morphology and may prevent neuroma formation.
	Yi et al. (71)	2018	Rat	PRGD/PDLLA conduit results in more normal nerve morphology, decreases collagen deposits, and decreases autotomy	
	Bolleboom et al. (72)	2018	Rat	Y-tube conduit with autograft prevents neuroma at 12 weeks after sharp transection	
	Zhou et al. (69)	2019	Rat	Aligned nanofiber conduit results in more organized nerve fibers, lower weight ratio of neuromas, and decreased autotomy	
	Onode et al. (70)	2019	Rat	Bioabsorbable polymer nerve conduit results in decreased autotomy and prevented neuroma formation	
Nerve coaptation	Gonzalez-Darder et al. (76)	1985	Rat	Centrocentral anastomosis reduces neuroma size and incidence of autotomy	Nerve coaptation may result in smaller neuromas but does not prevent axonal regeneration
	Low et al. (73)	1999	Rat	End-to-side anastomosis of transected nerves results in smaller masses and contained regenerating nerve tissue	
	Al-Qattan et al. (74)	2000	Rat	End-to-side anastomosis results in epineurial continuity, does not prevent axonal regeneration after nerve transection	
	Belcher et al. (77)	2000	Human	Centro-central union after digit amputation leads to decreased tenderness and sensation after amputation	
	Aszmann et al. (75)	2003	Rat	End-to-side neurorraphy results in sensory axonal organized regeneration	
	Economides et al. (18)	2016	Human	Peripheral nerve coaptation with collagen nerve wrapping prevents neuroma (0% vs. 36%) and phantom limb pain (0% vs. 64%) when compared to traction neurectomy	
Targeted muscle reinnervation (TMR)	Bowen et al. (84)	2019	Human	TMR prevents the development of symptomatic neuroma	TMR has shown great efficacy in preventing post-amputation pain and neuroma formation
	Valerio et al. (85)	2019	Human	TMR decreases residual limb pain and phantom limb pain	
	Alexander et al. (87)	2019	Human	TMR results in decreased frequency and intensity of neuroma symptoms in oncologic amputees	
	Chang et al. (86)	2021	Human	TMR decreases residual limb pain (14% vs. 57%), phantom limb pain (19% vs. 47%), increases ambulation (91% vs. 70%), and decreases opioid use (6% vs. 26%) compared to traction neurectomy	
Regenerative peripheral nerve interface (RPNI)	Kubiak et al. (92)	2019	Human	RPNI prevents symptomatic neuroma (0% vs. 13%) and decreases phantom limb pain (51% vs. 91%) compared to controls	RPNI may prevent neuroma formation and decrease post-amputation pain
TMRpni	Kurlander et al. (93)	2020	Human	Description of technique in limited cohort results in prevention of residual limb or phantom pain	Combination of two successful methods to provide mechanisms for organized regrowth

### Isolating the Site of Potential Neuroma Formation

#### Implantation of Proximal Nerve Stump into Adjacent Tissue

Implantation of the proximal nerve stump into adjacent tissue is a well-studied method for prevention of neuroma at time of nerve injury. First described in 1920, a severed nerve resulting from war injury was “driven into bone” preventing neuroma formation (24). Boldrey et al. later described the implantation of the proximal stump of transected ulnar nerve into the humerus in a canine model, resulting in significantly reduced size of neuroma (25). Proximal nerve stumps have also been implanted into adjacent muscle in baboons (26), monkeys (27), and rats (28–30), resulting in decreased size of neuroma and more organized nerve fibers. Nerve implantation into vein has also been studied in animal models, with improved results when compared to implantation into muscle. Prasetyono et al. found that while the size of neuroma from nerves implanted into muscle was decreased when compared to control nerves, implantation into vein resulted in the absence of neuroma without venous thrombus (29), results that have been replicated in additional rat studies (31, 32). While implantation of nerve into adjacent muscle will not result in re-innervation without first denervating the muscle, the implantation may provide the protective quality of isolating a potential neuroma from stimulation, leading to a decrease in severity of symptoms from the neuroma itself. Overall, implantation into adjacent tissue is a well-studied method that may not be successful in preventing neuroma formation, but often results in more organized nerve architecture and smaller neuroma size. More recently, successful peripheral nerve implantation into denervated muscle and the subsequent reinnervation of the muscle by transferred nerve has led to the advent of new techniques to prevent neuroma formation, including targeted muscle reinnervation and regenerative peripheral nerve interface (*vide infra*), rendering simple implantation of a transected nerve into adjacent muscle outmoded.

### Suppressing Axonal Regeneration

#### Pharmacologic

Pharmacologic inhibition of axonal regrowth has been attempted at the time of nerve injury since the early 20^th^ century. Chemical agents such as ethyl alcohol, phenol, and formaldehyde have been injected into nerves during surgery to prevent neuroma. In 1942 Guttman et al. studied the effects of an array of substances injected into transected tibial nerves of rabbits (33). Formaldehyde (20%) and gentian violet (1%) were found to be superior in prevention of neuroma for durations of up to one year, while absolute alcohol penetrated nerve tissue poorly and was effective for only two months. Formalin (5%), alcohol (80%), and phenol also inhibited neuroma formation in canines when injected directly into proximal nerve stumps (34). Ricin and doxorubicin have been investigated to prevent neuroma formation via retrograde transport of toxin resulting in cell body death (35–37). Ricin results in cell body death in 14–28 days and inhibits neuroma formation, although its clinical use is not recommended due to its extreme toxicity (36). Doxorubicin has similar but less pronounced effects, with infrequent cell body death while inhibiting axonal growth. Pharmacologic inhibition of nerve growth factor (NGF) was demonstrated by Kryger et al. in 2001 (38). As NGF is essential for nervous system development and nerve regeneration after peripheral injury, trkA-IgG (a highly specific anti-NGF protein) was studied for prevention of traumatic neuroma in rats. Results were mixed, as trkA-IgG produced a decrease in neuroma formation (38% of treatment rats vs. 80% of control rats) but did not completely inhibit axonal regeneration. While multiple pharmacologic agents have been shown to decrease the size and frequency of neuroma, a reliable method that is not systemically toxic has yet to be demonstrated.

#### Synthetic Capping

Placing a cap of a synthetic material on the terminal end of a transected nerve as a physical barrier to nerve regeneration was an early attempted method to prevent neuroma. Metal, plastic, rubber and resin were some of the materials studied, with varying success. In 1945 White et al. rolled thin sheets of tantalum around the nerve stump and crushed the open metallic end closed to prevent axonal outgrowth and neuroma formation (39). Edds et al. successfully inhibited neuroma formation with synthetic caps of 10% methyl methacrylate resin in acetone. It was hypothesized that the outer plastic shell of the cap hardened quickly, hindering the evaporation of acetone thus leading to nerve protein death and fixation, and the hard plastic shell prevented axonal outgrowth from any remaining viable nerves (40). In 1977 Swanson et al. described the successful use of silicone nerve caps in prevention of amputation neuroma in rabbits (41). Importantly, it was found that cap diameter must be close to the diameter of the severed nerve, as neuroma formed when the cap was significantly wider than the nerve allowing escape of regenerating axons. This silicone capping method was later utilized in treating symptomatic neuromas in humans and proved successful in relieving symptoms in 83% of patients (41). While many synthetic capping modalities have shown promise in animal models, they have fallen out of favor due to the risk of foreign body reaction, and capping has more recently been studied utilizing biodegradable or autologous materials.

#### Epineurial Closure and Fascicle Cauterization

Cauterization of the amputation nerve stump was first described by Bate in 1944, inspired by the absence of pain after electrical desiccation of skin tumors (42). Four patients underwent bipolar cauterization of nerve stumps during lower extremity amputation until the nerve had a dry, yellow, coagulated appearance. While the patients reported greater post-operative pain than usual after amputation, this pain ceased after four to six weeks. Battista et al. utilized this concept in their study of fascicle ligation and cauterization to prevent neuroma formation (43) and found that while a cut nerve with ligated fascicle had more extensive neuroma formation than the cut nerve control, cauterization of the fascicle significantly decreased the amount of regenerating nerve fibers. Closure of the epineurium to prevent axonal regrowth has also been explored. Martini et al. successfully prevented neuroma formation in rat sciatic nerves by closing the epineurial sleeve with histoacryl glue over a shortened nerve stump (44). Notably, direct application of histoacryl glue on the end of a cut nerve (no shortening of fascicles) did not prevent formation of neuroma, present in 68% of examined specimens. Rahimi et al. utilized radiofrequency from the Ellman Surgitron to seal the epineurial cap on a proximal nerve stump, however this method proved only partially successful as 44% of treated subjects exhibited bulbous neuromas at six months postoperatively, compared to 77% of control subjects (45). While epineurial closure and fascicle ligation has been shown to decrease the frequency of neuroma formation, this technique has not reliably demonstrated an ability to prevent neuroma formation and is not currently used in the clinical setting.

#### Laser Photocoagulation

Based on the hypothesis that laser neurectomy would “seal” the severed end of a transected nerve via photocoagulation of collagenous tissue, thus preventing axonal regenerative outgrowth, multiple types of lasers have been studied for prevention of neuroma. Laser transection has been well-studied with mixed results, varying with laser type and power. CO_2_ laser energy (infrared, 10.6 µm wavelength) has been widely studied in neurectomy, having the ability to seal tissue at low-power density and cut tissue at high-power density (46). However, the CO_2_ laser has not proven effective with studies showing no benefit vs. sharp transection (47), an inability to prevent distal axonal regeneration (48), and generally inconclusive results (49, 50). The neodymium:yttrium aluminum garnet (Nd:YAG) laser (1064 nm) was proposed as a superior tool for laser neurectomy due to a greater ability to penetrate deeply in tissue (51), and multiple studies have demonstrated its efficacy in decreasing neuroma formation after neurectomy. Menovski et al. reported superior results with 12 W average power (51) when compared to another study utilizing 10 W laser power (52). However, the Nd:YAG laser proved more efficacious at both 10 and 12 W when compared to sharp transection in preventing neuroma. Although the CO_2_ laser has proven inadequate, the Nd:YAG laser may prove more successful at higher power, however neither modality is currently used in the clinical setting.

#### Autologous Capping

**Figure 1:**
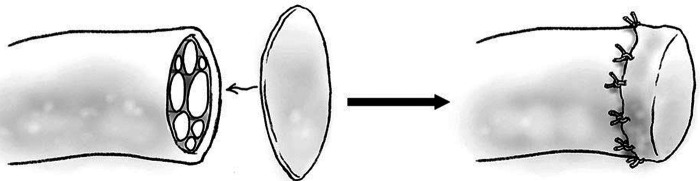
Diagram of epineurial cap.

Capping the proximal nerve stump with autologous material at time of nerve injury is a well-studied method to prevent formation of traumatic neuroma. Autologous tissue, such as vein or epineurium, is promising as it would not provoke an immune response and can be harvested from an amputated limb at no additional cost or morbidity to the patient. Nerve capping with epineurium has been well studied in rats, demonstrating decreased neuroma formation in transected sciatic nerves with reduced fibrosis and less abnormal nerve architecture (53, 54). Epineurial capping (Figure 1A) has also been trialed in humans and has proven effective, resulting in significantly decreased nerve pain post-amputation when compared to epineurial ligature or flaps (Figure 1B) (55). Nerve capping with autologous vein has also demonstrated an ability to prevent neuroma and preserve normal nerve architecture in rats (56). Vein is a promising autologous tissue for capping as it is easily harvested during amputation, may be dilated to fit over a nerve stump, and does not involve risk of venous thrombosis. Nerve capping with autologous material is a viable option for the microsurgeon to consider in order to decrease neuroma formation and nerve pain after peripheral nerve injury, although potential limitations may be a potential donor site for harvest of autologous tissue, degradation of the tissue and potential failure of the nerve cap.

#### Biomaterial Capping

Capping the proximal nerve stump with numerous biocompatible materials has been investigated in rats as a potential method to inhibit axonal regeneration after nerve transection. Biomaterials can prove stronger and more durable than autologous materials used in nerve capping and do not require an extra donor site as there is no harvesting of tissue. Microcrystallic chitosan has shown promise in inhibiting the formation of neuroma when applied as a gel-like cap on a transected sciatic nerves in rats, as Marcol et al. demonstrated a stump neuroma in only 42% of treatment rats compared to 100% of control rats (57). Additionally, when a neuroma did form on a nerve treated with microcrystallic chitosan, it was significantly smaller than those in control rats with significantly decreased extraneural fibrosis. Seprafilm®, (chemically modified hyaluronic acid and carboxymethylcellulose; Genzyme Corporation, Cambridge, MA) commonly utilized surgically to reduce intra-abdominal adhesions, has also inhibited axonal regeneration when applied directly to the end of a transected nerve (58). Nerve caps composed of porcine small intestinal submucosa (pSIS), a biologic material that remodels into a permanent soft tissue layer when implanted, has shown promise as a terminal nerve cap (59). Tork et al. showed pSIS nerve caps placed on transected rat tibial nerves resulted in decreased aberrant axonal regeneration, decreased neuroma formation and decreased pain. Preclinical rat studies investigating acellular nerve allografts (ANAs) to repair nerve gaps of various lengths demonstrated that axons, while capable of spanning short (<3 cm) ANAs to reinnervate distal targets, ceased regenerating within long (>4 cm) ANAs, prompting studies of nerve capping with long ANAs. Hong et al. showed ANAs 2.5 cm in length or greater contained no regenerating axons in the most distal tissue region (60). Pan et al. demonstrated downregulation of regenerative and pain-related genes upstream, in addition to success inhibition of axonal regrowth at 5 months (61). Nerve capping with biomaterials remains a promising technique with successful preclinical studies in rats, but future large animal and human trials are warranted prior to clinical use.

### Providing a Conduit for Organized Regrowth

#### Nerve Conduit

**Figure 2:**
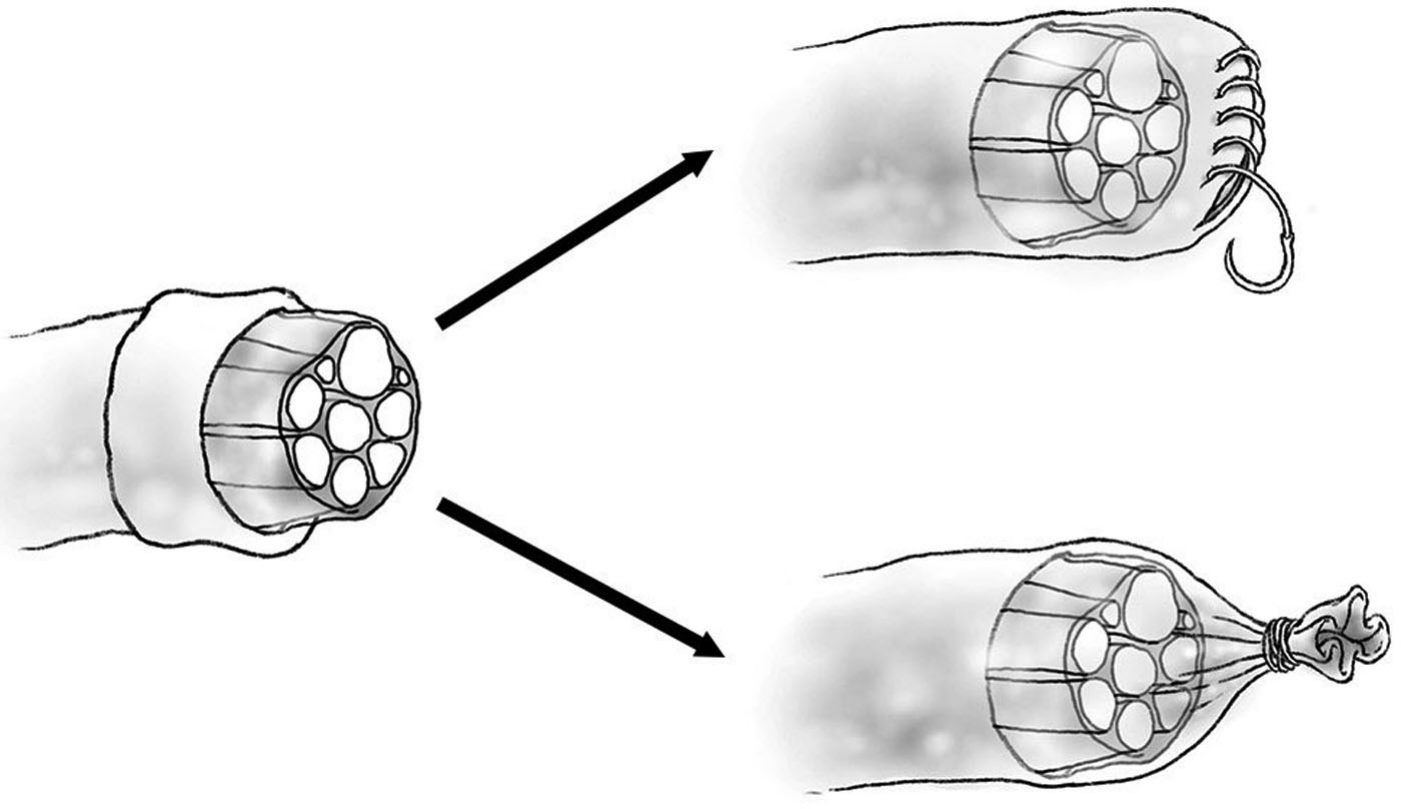
Diagram of epineurial flap (top) and epineurial ligature (bottom).

Placing a hollow tube, known as a conduit, on the terminal end of a proximal nerve to prevent neuroma first arose from attempts to bridge long gap nerve injuries. This concept was first described by Gluck in 1881, who bridged surgically created nerve gaps with bone, braided catgut and muscle and hypothesized that nerve regeneration may occur with parts of the nerve “climbing up the scaffold of the implanted foreign body as vine climbing a staff” (62). In 1891 Büngner described utilizing a segment of human brachial artery to bridge a canine sciatic nerve gap (63). In 1944 Weiss experimented with long gap nerve reunion utilizing tantalum foil tubes (64), building on prior research using freeze-dried artery in the same model (65). Nerve reunion was performed by placing transected peroneal or tibial nerve into tantalum cuffs. At five weeks the tantalum tubes were removed from the injured nerve site, revealing the absence of branching or straying nerve fibers at the level of injury without neuroma. It was hypothesized that due to the linear nature of the tube regenerating nerve fibers maintained a more orderly course, an important concept in prevention of neuroma. Recently, multiple studies of various conduit materials have been performed in a rat sciatic nerve model, including collagen (66), nanofiber (67–69), and bioabsorbable conduits (70, 71). These studies have all produced similar results, including decreased autotomy, decreased size of neuroma and improved nerve morphology with the use of proximal nerve conduit. In the setting of amputation, a nerve conduit may provide a contained path for regenerating axons to traverse, and over time this regeneration will cease before axons reach the end of the conduit, preventing the formation of a bulbous neuroma. Bolleboom et al*.* describe a novel 3D-printed Y-tube, with a transected rat sciatic nerve inserted into the single arm of the conduit and a peroneal nerve graft connecting the Y arms of the conduit (Figure 2) (72). At 12 weeks, regenerating axons had grown into both ends of the autograft, analogous to a centrocentral coaptation scenario, without evidence of neuroma. Nerve conduits are a promising mechanism for prevention of neuroma formation at time of injury and may warrant future study before clinical use in humans.

#### Nerve Coaptation

**Figure 3:**
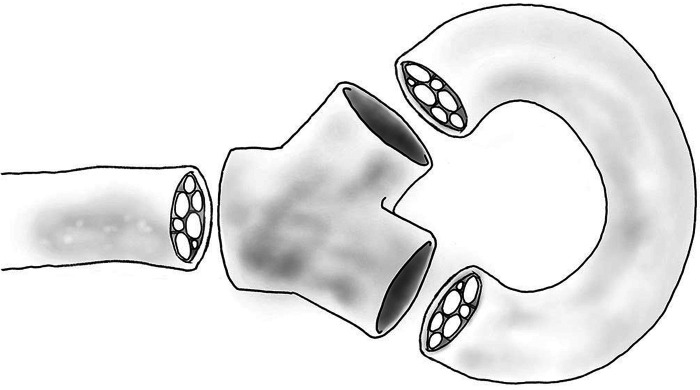
Diagram of open-ended Y-tube with autograft.

Following peripheral nerve injury where the distal nerve stump is unavailable, the end of the injured proximal stump can be coapted to the side of an adjacent intact nerve, the end of an additional transected nerve, or coapted to fascicles from the same transected nerve (after fascicular dissection). End-to-side nerve coaptation, which may require creation of an epineural window prior to coaptation, theoretically provides a conduit for axonal regrowth via the recipient nerve (20). The technique has demonstrated mixed results in both rats and humans. Low et al. transected the lateral branch of the sciatic nerve and coapted the proximal stump into the intact main sciatic nerve, resulting in regenerating nerve fibers being contained within the anastomosis (73). Electron microscopy of the coapted nerves revealed the absence of the large abnormal myelinated fibers that were seen in control nerves. Al-Qattan et al. demonstrated positive results when the transected end of rat tibial nerve was coapted into nearby peroneal nerves; the implanted nerve healed into peroneal nerve with epineurial continuity (74). This technique was also successful when implanting the proximal rat saphenous nerve stump into tibial nerve, with absence of neuroma and implanted saphenous nerve axons penetrating the tibial perineurial sheath (75). In 2016 Economides et al. demonstrated success in humans with coaptation of the common peroneal nerve to tibial nerve at time of amputation. Six months after surgery patients who underwent nerve coaptation reported decreased pain, had strikingly lower rates of formation of neuroma (0% vs. 54%) and decreased phantom limb pain (0% vs. 63%) when compared to those who underwent traction neurectomy (18). This technique may provide a defined pathway for regenerating nerves to follow, as demonstrated by penetration of perineurial sheath and healing of transected nerve into the receptive nerve. Either through intraneural fascicular coaptation (Figure 3) or direct end-to-end neurorrhaphy of adjacent distal nerve endings, a technique known as centrocentral nerve coaptation has been applied successfully efficacy in preventing neuroma formation in rats (76) and has also resulted in decreased tenderness to palpation after finger amputation in humans (77). The technique has also been used for treatment of painful neuroma in lower extremities (78, 79) and intercostal nerves (80). An additional elegant example of a nerve coaptation technique that can prevent neuroma formation is the pedicled ulnar nerve transfer, a two stage technique also known as the St. Clair Strange procedure, which allows for successful bridging of large median nerve defects in humans (81). In this procedure, the proximal median nerve stump is coapted into the proximal ulnar nerve stump, allowing for median nerve axonal regeneration and retrograde growth of nerve fibers into the proximal ulnar nerve stump. When sufficient retrograde regeneration has occurred into the proximal ulnar nerve stump (as assessed by Tinel’s sign), the ulnar nerve to be transferred to the distal median nerve target is pedicled and transferred distally. The St. Clair Strange procedure has resulted in the return of protective sensibility without formation of a neuroma after long gap nerve injury. By providing a pathway for axonal regeneration, a painful stump neuroma can possibly be prevented with nerve coaptation, although this technique has fallen out of favor due to higher rates of neuroma formation, as well as the advent of newer techniques that provide a conduit for organized regrowth for the transected nerve.

#### Targeted Muscle Reinnervation

**Figure 4:**
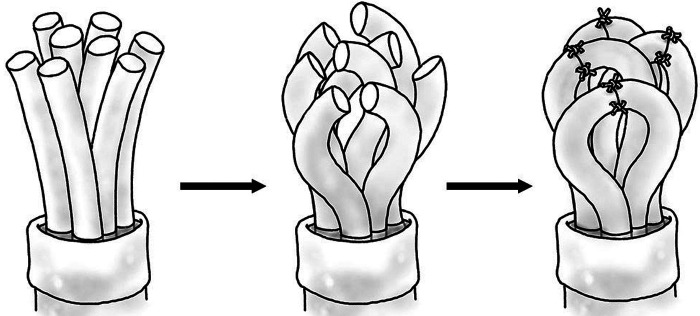
Diagram of fascicular end-to-end coaptation.

Targeted muscle reinnervation (TMR) was first described by Kuiken et al*.* in 2004 as a surgical technique to transfer intact proximal nerve stumps after amputation to alternative muscle sites to improve myoelectric prosthesis control (82). This technique involves the transfer of the proximal sensory or mixed sensory/motor nerves to motor nerves of residual intact muscles for the purpose of generating reliable EMG signals (Figure 4). The reinnervated residual muscle then receives motor commands from the coapted peripheral nerve and provides EMG signals for prosthetic control. While successful with regards to prosthetic control, TMR has also proven to have the secondary benefit of preventing the formation of neuroma in these transected mixed motor and sensory peripheral nerves that are coapted to the smaller motor nerve branches of the residual muscles. Souza et al*.* reported twenty-six patients over ten years that had TMR performed for the purpose of prosthesis control (83). While fifteen patients had symptomatic neuroma pain prior to TMR, 93% of these patients noted complete resolution of nerve pain after undergoing TMR, and none of the eleven patients that were initially pain free prior to TMR subsequently developed a symptomatic neuroma. Bowen et al*.* reported twenty-two human patients that underwent below knee amputation, none of whom developed symptomatic neuromas post-operatively (84). Additionally, phantom limb pain was present in only 13% of patients at six months, a significant improvement when compared to institutional controls. Valerio et al*.* reported a multi-institutional cohort study with fifty-one patients undergoing TMR at time of major limb amputation, demonstrating patients that underwent TMR reported significantly lower levels of both neuroma-related residual limb pain and phantom limb pain (85). In a 2021 landmark cohort study comparing 100 patients who underwent below knee amputation (BKA) with traction neurectomy and muscle implantation against 100 patients who underwent BKA with TMR, TMR was found to result in more pain-free patients (71% vs. 36%), decreased residual limb pain (14% vs. 57%), decreased phantom limb pain (19% vs. 47%), decreased opiate use by TMR patients (6% vs. 26%), and more TMR patients were ambulatory (91% vs 71%) (86). These results have been replicated in the setting of amputation for cancer diagnoses, demonstrating decreased post-operative incidence of neuroma, residual limb pain, and opioid use when compared to control amputees (87). One mechanism behind the success of TMR in preventing neuroma pain is the purpose it provides for the transected peripheral nerve, which may prevent the haphazard axonal regeneration commonly seen after neurectomy. TMR requires a surgeon familiar with peripheral nerve anatomy and trained in nerve coaptation, and requires additional operative time compared to a typical lower limb amputation. With minimal additional morbidity inflicted upon the patient, TMR is a promising modality that may soon be performed routinely at time of amputation to prevent symptomatic neuroma.

#### Regenerative Peripheral Nerve Interface

**Figure 5:**
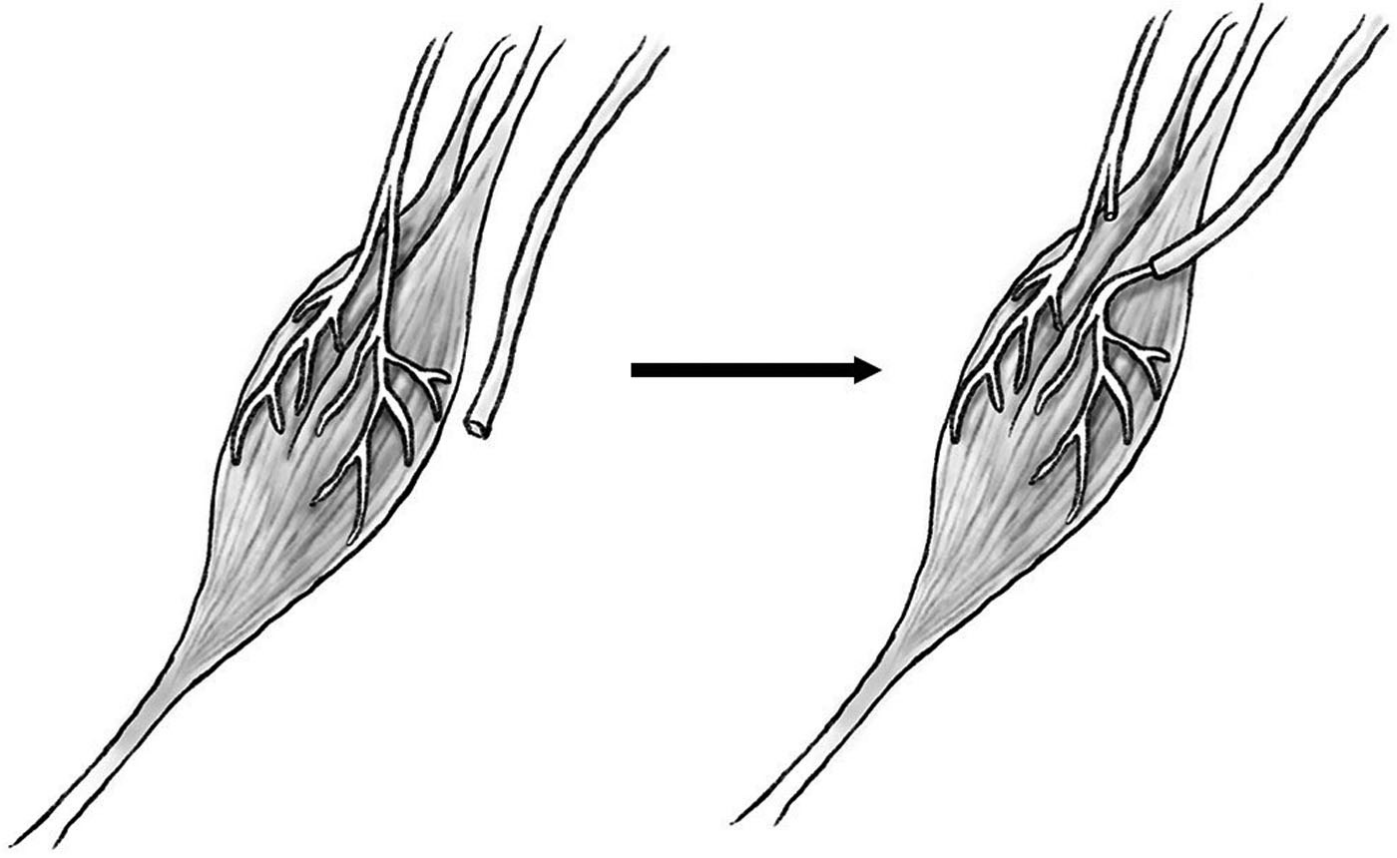
Diagram of transected proximal nerve stump (left) transferred to motor nerve of muscle (right).

Regenerative peripheral nerve interface (RPNI), like TMR, was initially developed as a mechanism to generate EMG signals from divided peripheral nerves to use as control signals for a prosthetic limb (88), but has been found similarly to have the secondary benefit of preventing neuroma formation in the divided nerve. RPNI involves a free muscle graft (frequently vastus lateralis) that is neurotized by a transected peripheral nerve (Figure 5), with the muscle graft amplifying bioelectric signals and housing an electrode for signal transduction to a neuroprosthetic limb (89). The denervated free muscle graft undergoes revascularization and becomes a target for the regenerating axons of the transected peripheral nerve, resulting in the formation of new neuromuscular connections instead of a neuroma (90). RPNI has been utilized for treatment of symptomatic neuroma (91) as well as prophylactically at time of initial amputation to prevent formation of neuroma (92). Kubiak et al. reported forty-five patients who underwent prophylactic RPNI at time of index amputation, with 0% of RPNI patients developing symptomatic neuroma compared to 13% of control patients (92). This study also demonstrated a significant reduction of phantom limb pain in RPNI patients (51% vs. 91%) when compared to controls. Although RPNI required an additional 62 min of operative time on average when compared to control operations, patients who underwent RPNI spent six fewer days in hospital (11.7 ± 21.2 vs 17.6 ± 24.3) and had fewer overall rates of complications (31% vs 56%) in addition to the reduction in pain and neuroma formation. Because free muscle grafts can be harvested from amputated tissue and there is no sacrifice of intact donor nerves (90), RPNI is a low morbidity option for prevention of neuroma formation that has demonstrated promising results in limited human trials.

**Figure 6:**
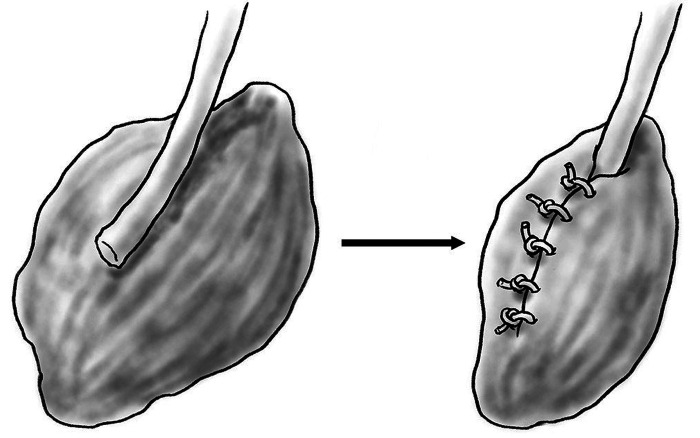
Diagram of transected peripheral nerve wrapped with free denervated muscle graft.

#### TMRpni

Drawing off of the success of both TMR and RPNI to prevent neuropathic pain at time of amputation, the advent of a technique that utilizes principles of both TMR and RPNI has been described by Kurlander *et. al*. Coined TMRpni, the technique involves executing a TMR nerve coaptation followed by wrapping the coapted nerve with an autologous free muscle graft, as would be performed in RPNI (93). This technique could prove beneficial in situations where standard TMR is less than ideal, such as when a large donor nerve is coapted to a smaller motor target. If the donor nerve contains more axons than are available in the recipient motor nerve, donor axonal escape is possible and could lead to formation of neuroma. In TMRpni, the free muscle graft around the coaptation provides a target for donor axons that may have escaped, preventing aberrant axonal regrowth and neuroma formation. While the authors only describe outcomes in one patient with an above-knee amputation, the patient is without residual limb or phantom pain four months after the procedure. TMRpni requires minimal additional operative time and costs when compared to standard TMR and RPNI and is potentially a mechanism to improve upon the already demonstrated success of TMR and RPNI.

## Discussion

Painful neuroma is a frequent sequela of traumatic peripheral nerve injury during limb amputation that surgeons have attempted to prevent with a wide array of surgical methods for over 100 years. The common theme of these surgical attempts has been prevention of disorganized axonal regrowth from the proximal nerve stump. There are a variety of methods currently studied or used clinically at time of nerve injury to prevent neuroma formation, with some relying on more technical skill and peripheral nerve expertise than others. Older methods of preventing axonal outgrowth (pharmacologic inhibition, synthetic capping, laser transection) are becoming less clinically relevant, due to unpredictable and graded results. Biomaterial capping has shown positive results in animal studies but requires further investigation in humans to examine viability and efficacy. Newer methods giving “purpose” to the regenerating axon (TMR, RPNI, TMRpni) have demonstrated promising results, but lack more than a handful of clinical series and require a surgeon experienced in peripheral nerve surgery. Painful symptomatic neuroma after peripheral nerve injury has been a difficult problem for the surgeon since first described in 1811, but much progress has been made towards routine prevention of this complication at time of injury.
